# Renal abnormalities among children with sickle cell conditions in highly resource-limited setting in Ghana

**DOI:** 10.1371/journal.pone.0225310

**Published:** 2019-11-19

**Authors:** Enoch Odame Anto, Christian Obirikorang, Emmanuel Acheampong, Eric Adua, Sampson Donkor, Bright Oppong Afranie, Matthew Ofori, Emmanuel Akomanin Asiamah, Evans Asamoah Adu

**Affiliations:** 1 Department of Molecular Medicine, School of Medicine and Dentistry, Kwame Nkrumah University of Science and Technology, Kumasi, Ghana; 2 School of Medical and Health Sciences, Edith Cowan University, Joondalup, Western Australia, Australia; 3 Department of Medical Laboratory Technology, Royal Ann College of Health, Atwima-Manhyia, Kumasi, Ghana; 4 Department of Medical Laboratory Science, University of Health and Allied Sciences, Ho, Ghana; University of Oxford, UNITED KINGDOM

## Abstract

Sickle cell disease (SCD) is associated with progressive multi-organ failure especially, the brain and kidney and leads to high morbidity and mortality rate. The aim of this study was to determine the prevalence of renal abnormalities among children with SCD. This cross-sectional study recruited 212 sickling positive patients comprising of 96 Hb AS, 48 Hb SC, and 68 Hb SS phenotypes from the Pediatric Unit of Wassa Akropong Government Hospital, Wassa Akropong, Ghana. Early morning urine and venous blood samples were collected from each participant. Urinalysis was conducted and serum urea and creatinine levels were estimated. Estimate glomerular filtration rate (eGFR) was calculated using the Swartz equation. Classification of chronic kidney disease (CKD) was based on ‘The Kidney Disease: Improving Global Outcomes (KIDIGO)’ criteria. The mean age of the children were 7.90 years. Serum creatinine (p = 0.0310) and urea (p<0.0001) levels were significantly higher among Hb AS participants compared with Hb SS phenotype. The prevalent indicators of renal abnormalities were proteinuria (26.4%), urine granular cast (5.6%) and CKD (39.6%). Proteinuria, urine granular cast and CKD were most prevalent among Hb SS (47.1%, 11.8% and 73.5% respectively) compared with Hb SC (41.7%, 8.3%, and 45.8% respectively) and Hb AS (4.2%, 0.0%, and 14.5%) phenotypes, respectively. Sickle cell conditions were significantly associated with proteinuria (p<0.0001) and CKD (p = 0.0378). Children with Hb SS [aOR = 5.04, 95% CI (2.47–10.3); p<0.0001] and Hb SC [aOR = 3.14 95% CI (1.39–7.01); p = 0.0174] were at increased odds of developing CKD after adjusting for age, BMI and gender. Proteinuria and CKD are associated with sickle cell disease (Hb SC and Hb SS). Renal function should be routinely monitored for children with SCD.

## Introduction

Sickle cell disease (SCD) is a major genetic disorder; which occurs due to a mutation in the globin gene of hemoglobin [1, 2]. It affects millions of people worldwide, but it is particularly common among sub-Saharan Africans (SSA) [3]. The general prevalence of SCD in Ghana has remained at 2% and the trait 25% in the population [4]. According to Ohene-Frempong et al., [4] the prevalence rate of SCD in Ghana is 1.9% of all births per years and it is responsible for several premature deaths. The Ghana Health Service claims that SCD was the 37^th^ and 36^th^ cause of deaths in 2002 and 2003 respectively [5, 6]. High morbidity and mortality is common with the homozygous HbSS phenotype [7]. Even though clinical manifestation of SCD involves a wide array of symptoms, recurrent attacks of vaso-occlusive crisis (VOC) is the most common, which consequently stimulate defects in the renal medulla [8, 9].

Patients with sickle cell anaemia (Hb SS) or sickle cell trait (Hb AS) may present with several types of renal dysfunction as a result of chronic anaemia leading to hemodynamic changes; and by the consequences of vaso-occlusion especially in the renal medulla [10, 11]. The disruption of the distal nephron and medullary function leads to a reduction in renal concentrating capacity, urinary acidification, and impairment in potassium metabolism which are often observed in these patients [12, 13]. In addition, patients with SCD develop glomerulopathy which can worsen into renal insufficiency [14]. At the same time, pulmonary hypertension associated with SCD, leads to hemolytic anemias such as thalassemia, hereditary spherocytosis and paroxysmal nocturnal hemoglobinuria [15]. Taken together these complications, the risk of chronic kidney disease (CKD) or End Stage Renal Disease (ESRD) is certain among SCD patients [16].

Early detection and treatment is necessary to avoid renal complications. However, the risk factors for progression are not clearly elucidated, but significant albuminuria is a key factor in progression [17]. Acknowledging the limited resources available for managing the disease in most parts of tropical Africa, and the lack of accurate data to assess the impact of the SCD on public health in general, 95% of children born with the disease die before the age of 5 years [18]. The expensive and high-technological solutions to healthcare development in high income countries are not best suited to a developing country like Ghana. Thus, investigations of multi-organ damage are only conducted following an acute inlness or clinical manifestation of symptoms [4].

Aside the challenges in the management of children with SCD in resource-limited districts in Ghana, there is the need for ample data on renal dysfunctions as well as early detection to create awareness for routine check-ups. An earlier study has reported a CKD prevalence of 39.2% [17]. Using the KDIGO guidelines, the prevalence of CKD by haemoglobin phenotype reported by Ephraim et al., [17] were 40.8% and 30.8% for HbSS and HbSC, respectively. Therefore, this study determined the prevalence of renal abnormalities among children with SCD in a highly resource limited setting in Ghana.

## Materials and methods

### Study design and setting

This cross-sectional study was carried out at the Pediatric Unit of Wassa Akropong Government Hospital in the Wassa Amenfi East district of the Western Region of Ghana from August 2016 to September 2017.

### Study subjects

A total of 212 known sickle cell positive children comprising of 96 hemoglobin phenotype AS (HbAS), 48 hemoglobin phenotype SC (HbSC) and 68 hemoglobin phenotype SS (HbSS) patients aged five (5) to twelve (12) years. Sickling positive outpatients in a steady state, defined as patients without febrile symptoms or acute illness, were included in this study. Sickle cell positive patients with other diagnosed medical conditions such as viral hepatitis B or C, diabetes mellitus, tuberculosis and acute viral infections, urinary tract infections, joint inflammatory condition or any other chronic infections which may interfere with urine albumin dipstick analysis were excluded. Patients in vaso-occlusive crises were excluded from the study. Patients with dipstick results suggestive of either anaemic crises (increased bilirubin) or UTIs infections were excluded. Structured questionnaire was used to collect socio-demographic data of the study participants.

### Blood collection and biochemical analysis

A volume of three (3) mL of venous blood was taken from the subjects and transferred into gel separator tubes. Blood was allowed to clot and centrifuged at 1500 g for 3 minutes. Serum urea and creatinine levels were estimated enzymatically using BS3000M Semi Auto Chemistry analyser (SINNOWA Medical Science and Technology Co. Ltd.).

### Urine collection and biochemical analysis

Early morning urine was collected from each participant into a clean, sterile and leak-proof container. Urine protein was estimated using a highly sensitive semi-quantitative urine albumin dipstick. The Medi-Test Combi 10^®^SGL urinary strips was used for the dipstick analysis and all 10 parameters evaluated. Dipstick method was done once and results recorded without repetition of tests to confirm the abnormalities. A drop of decanted urine deposit was observed under microscope. The following were looked for: Epithelial cells, Pus cells, RBC’s and the various types of casts and crystal structures.

### Estimated glomerular filtration rate (eGFR) and CKD

The updated Schwartz equation [19] was used for calculating estimated glomerular filtration rate (eGFR) as follow

eGFR(ml/min/1.73m2)=0.413Xheight(cm)/serumcreatinine(mg/dl)

CKD was classified according to the Kidney Disease Improving Global Outcome (KDIGO); either decreased eGFR < 60 mL/min/1.73 m^2^ corresponding to stage 3–5 or evidence of kidney damage (albuminuria, or overt proteinuria) [20]. The various eGFR stages were defined as follows: Stage 1: ≥90 ml/min/1.73 m2 (Kidney damage with normal or increased eGFR); Stage 2: 60–89 ml/min/ 1.73m^2^ (Kidney damage with mildly decreased eGFR); Stage 3a: 45–59 ml/min/1.73 m^2^ (mild to moderately decreased eGFR); Stage 3b: 30–44 ml/min/1.73 m^2^ (moderate to severely decreased eGFR); Stage 4: 15–29 ml/min/1.73 m^2^ (severely decreased eGFR) and Stage 5: <15 ml/min/1.73 m^2^ (Kidney failure) [21].

### Anthropometry

Weight and height of children were measured using weighing scale and standiometer respectively. Age and sex-specific BMI percentile of children were categorized as underweight = <5^th^ percentile; normal weight = >5^th^, <85^th^ percentile; overweight = ≥85^th^ percentile and obese = >95^th^ percentile [22].

### Ethics approval and consent to participate

This study was approved by Ethical Review Board and Disease Control Unit of the Wassa Akropong Health Directorate and the Management of the Hospital. A written informed consent was obtained from the children as wells as legally authorized representative of children and confidentiality was maintained. Respondents were assured of confidentiality. In addition, the privacy rights of human subjects were observed. This study was carried out in accordance with the Code of Ethics of the World Medical Association (Declaration of Helsinki).

### Statistical analysis

The data collected was entered into Microsoft Excel and analyzed in STATA version 14. Data was presented in frequency (proportion) for categorical variables and mean standard deviation (SD) for continuous variables. Association between categorical variables was performed using Chi-square test. Comparison between three independent continuous variables was performed using One-way ANOVA followed by Tukey Test for multiple comparisons. Logistic regression model was performed to identify the risk of Hb Phenotype developing CKD. The level of significance was set at p<0.05 for all statistical comparisons.

## Results

The average age of the children were 7.9 years. Overall, a higher proportion of participants were 7–8 years (34.9%), followed by 5-6years (28.3%), 9–10 years (21.7%) and 11–12 years (15.1%). There were more boys compared to girls (55.7% vs. 44.3%). The proportion distribution of Hb phenotype between boys and girls did not show significant differences (p-value = 0.140). Children with Hb phenotype SS had significantly reduced weight and BMI compared with Hb AS (p<0.05). A higher proportion of children with Hb phenotype SS were underweight (73.5%). BMI status was significantly associated with Hb phenotype (p = 0.001) [Table 1].

**Table 1:** General characteristics of study participants.

Characteristics	Total (N = 212)	Hb variant			p-value
		AS (N = 96)	SC (N = 48)	SS (N = 68)	
**Age (years) (Mean ± SD)**	7.90 ± 2.2	7.39 ± 2.0	8.50 ± 2.1	8.206 ± 2.3	0.074
**Age Group (year) n (%)**					0.210
5–6	60(28.3)	36(37.5)	6(12.5)	18(26.5)	
7–8	74(34.9)	32(33.3)	22(45.8)	20(29.4)	
9–10	56(21.7)	18(18.8)	8(16.7)	20(29.4)	
11–12	32(15.1)	10(10.4)	12(25.0)	10(14.7)	
**Gender n (%)**					0.140
Boys	118(55.7)	52(54.2)	20(41.7)	46(67.6)	
Girls	94(44.3)	44(45.8)	28(58.3)	22(32.4)	
**Height (cm) (Mean ± SD)**	135.00 ± 0.1	134.00 ± 0.1	135.00 ± 0.1	134.00 ± 0.1	0.871
**Weight(kg) (Mean ± SD)**	26.12 ± 1.85	26.24 ± 1.96	24.41 ± 1.91	23.83 ± 1.97*	**0.038**
**BMI(kg/m**^**2**^**)**	18.48 ± 1.06	19.58 ± 1.07	18.08 ± 1.13	17.78 ± 0.98*	**0.041**
**BMI percentile**					**0.001**
Under weight(<5^th^)	102(48.1)	32 (33.3)	20(41.7)	30(73.5)	
Normal weight(5^th^-84^th^)	110(51.8)	64 (66.6)	28 (58.3)	18(26.5)	

Values are presented as frequency (proportion), Mean ± SD (standard deviation).

* indicate significant compared to hemoglobin Phenotype AS (HbAS) group.

BMI: body mass index; Hemoglobin Phenotype SC (Hb SC); Hemoglobin Phenotype SS (HbSS)

Table 2 shows the prevalence and association between measures of renal abnormalities and sickle cell conditions. The overall prevalence of proteinuria was 26.4%. The prevalence was higher among patients with Hb SS (47.1%) followed by Hb SC (41.7%) and Hb AS (4.2%). There was a significant association between proteinuria and SCDs (p-value = 0.0004). The overall prevalence of urine granular cast was 5.6%. The prevalence was slightly higher among Hb SS participants (11.8%) compared with Hb SC (8.3%). No granular cast was observed for Hb AS participants. Using the KIDIGO criteria, the overall prevalence of CKD was 39.6%. The highest prevalence of CKD was observed among patient with Hb SS phenotype (73.5%) followed by Hb SC (45.8%) and Hb AS participants (14.5%). There was a significant association between CKD and sickle cell conditions (p = 0.0378).

**Table 2:** Prevalence and association between renal abnormalities and sickle cell conditions.

Characteristics	Total (N = 212)	Hb variant			p-value
		AS (N = 96)	SC (N = 48)	SS (N = 68)	
**Urine Protein (albumin)**					**0.0004**
Negative	156(73.5)	92(95.8)	28(58.3)	36(52.9)	
1+	14(6.7)	2(2.1)	6(12.5)	6(8.8)	
2++	20(9.4)	2(2.1)	8(16.7)	10(14.7)	
3+++	22(10.3)	0(0.0)	6(12.5)	16(23.5)	
**Total proteinuria**	**56(26.4)**	**4(4.2)**	**20(41.7)**	**32(47.1)**	
**Granular Cast**					N/A
NIL	200(94.3)	96(100.0)	44(91.7)	60(88.2)	
1+	2(0.9)	0(0.0)	2(4.2)	0(0.0)	
2++	6(2.8)	0(0.0)	2(4.2)	4(5.9)	
3+++	4(1.9)	0(0.0)	0(0.0)	4(5.9)	
**Total Cast**	**12(5.6%)**	**0(0.0)**	**4(8.3)**	**8(11.8)**	
**KIDIGO Criteria**					
**CKD, n (%) eGFR**					**0.0378**
Stage 1: ≥90 + albuminuria	24(11.3)	4(4.2)	6(12.5)	16(23.5)	
Stage 2: 60–89 + albuminuria	16(7.5)	2(2.1)	4(8.3)	10(14.7)	
45–59 ml/min/1.73 m^2^	30(14.2)	4(4.2)	12(25.0)	14(20.5)	
30–44 ml/min/1.73 m^2^	14(6.6)	2(2.1)	2(4.2)	10(14.7)	
**Overall CKD, n (%) eGFR**	**84(39.6)**	**14(14.6)**	**22(45.8)**	**50(73.5)**	
**No CKD**	**128(60.4)**	**82(85.4)**	**26(54.2)**	**18(26.5)**	

Values are presented as frequency (proportion). N/A: not applicable; CKD: chronic kidney disease; KDIGO: The Kidney Disease: Improving Global Outcomes

Children with Hb SS phenotype were at 5 times increase odds of developing CKD [aOR = 5.04, 95% CI (2.47–10.3); p<0.0001] while Hb SC children were at 3 times increased odds of developing CKD [aOR = 3.14 95% CI (1.39–7.01); p = 0.0174] after adjusting for age, BMI and gender [Table 3].

**Table 3:** Logistic regression analysis of sickle cell condition and it association with CKD.

	CKD		cOR	p-value	aOR	p-value
	CKD (N = 84)	No CKD (N = 128)				
**Hb Phenotype**						
AS	14(16.7)	82(65.1)	1 (reference)			
SC	22(26.2)	26(20.6)	4.95(1.59–15.42)	0.0080	3.14(1.39–7.01)	0.0174
SS	50(59.5)	18(14.2)	16.3(5.38–49.18)	<0.0001	5.04(2.47–10.3)	<0.0001

aOR: Age, BMI, gender adjusted odds ratio; cOR: crudes odds ratio

**Fig 1:**
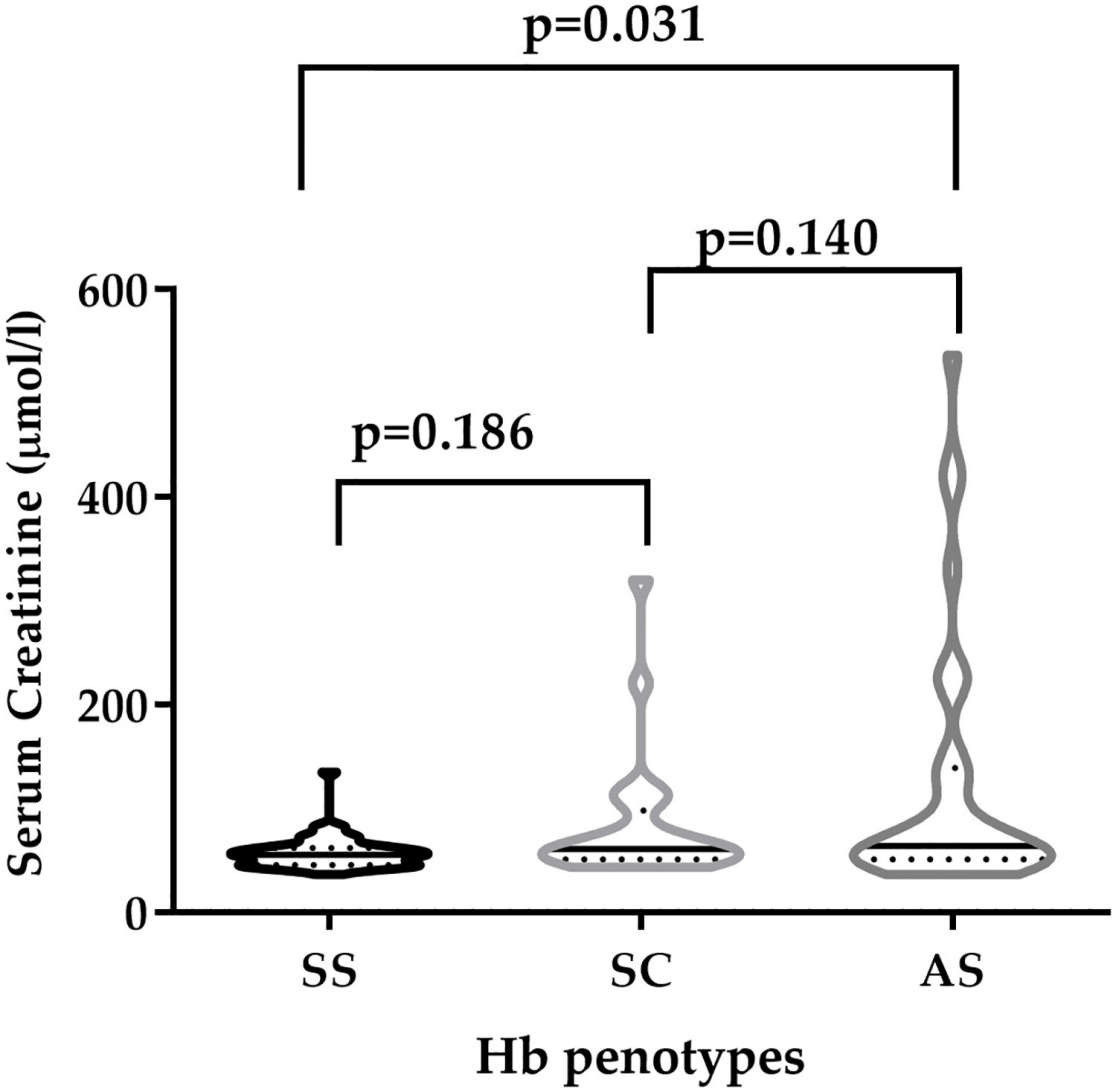
Serum creatinine levels stratified by Hb phenotype.

Fig 1 depicts the serum creatinine levels compared among Hb phenotype; Serum creatinine levels were significantly lower among Hb SS participants compared to their Hb AS counterpart (p = 0.0310). However, there were no statistically significant difference between: Hb AS and Hb SC (p = 0.1862); and Hb SC vs. Hb SS (p = 0.1402).

**Fig 2:**
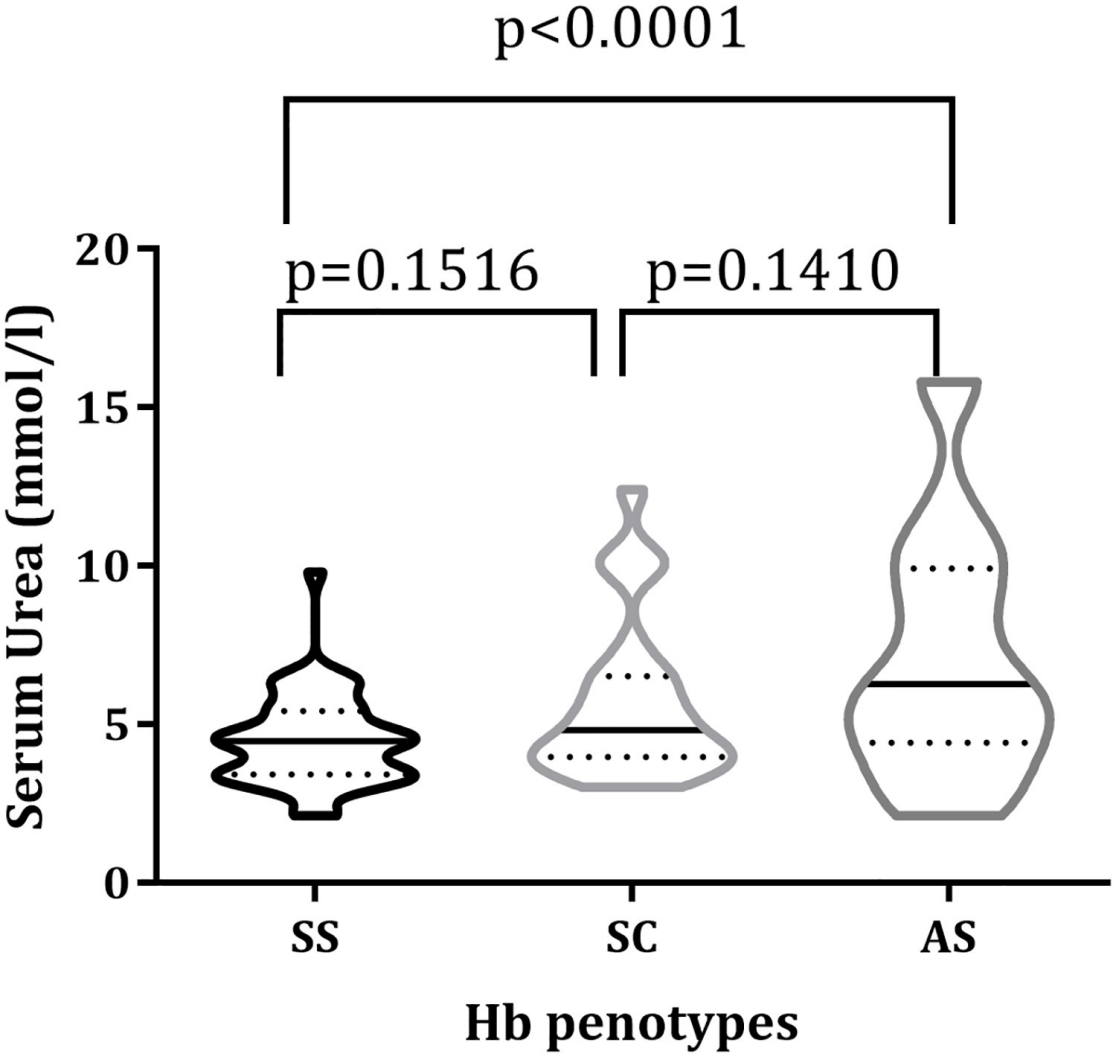
Serum urea levels stratified Hb phenotypes.

Fig 2 also shows the serum levels of urea compared among Hb phenotypes; the levels of serum urea among Hb SS participants were higher compared to Hb AS (p<0.0001). Conversely, serum urea levels between: Hb AS and SC (p = 0.1516); and Hb SC and Hb SS (p = 0.1410), participants did not significantly differ.

## Discussion

Sickle Cell Disease (SCD) affects various organ systems in the body, and renal abnormalities are not an exception [12]. This study determined the prevalence of renal abnormalities among children with sickle cell conditions in a highly resource limited SCD clinic in Ghana using less expensive routinely available urinalysis and blood urea and creatinine measurements. The combined diagnostic performance of eGFR and albuminuria were used for CKD classification.

The characteristics of the study participants indicated, that 48.1% of the children were underweight and the highest prevalence (73.5%) was observed among children with HbSS phenotype. This finding is consistent with the reports of Osei-Yeboah, Rodrigues and Enweronu-Laryea [23] who reported 61.0% prevalence of malnutrition among children with SCD in Ghana. A study by Boadu, Ohemeng, and Renner [24] has reported similar findings in recent years. Whiles underweight could undermine the estimation of eGFR by decreasing creatinine levels [24], it is also a strong index for chronic renal failure [25, 26]. In Africa, the aetiology of malnutrition in CKD has been associated with the chronic complications such as frequent crises, hepatomegaly, renal failure, anaemia and persistent infections [27].

Proteinuria prevalence was high among children with SCD compared with those with the sickle cell trait (HbAS). A dipstick urinalysis findings by Osei-Yeboah and Rodrigues [28] among SCD children in an urban setting in Ghana, reported an isolated proteinuria prevalence of 2.8% which is significantly lower compared with our findings. However, studies Ephraim et al [17] using the observed a proteinuria prevalence of 39.2% among SCD population in an urban settlement in Ghana using the dipstick method. These reported inconsistencies can be associated with age, which was demonstrated by Osei-Yeboah and Rodrigues [28]. Also, the disparity in findings could be attributed to different population group used across different studies and other confounding factors. Proteinuria often develops in childhood thus preventive and treatment approaches for sickle cell nephropathy should be an emphasis of pediatric programs [29]

Proteinuria is a strong, independent predictor of renal abnormalities and failure [30]. In a prospective longitudinal study by Anigilaje and Adedoyin [31] in Nigeria showed that proteinuria is persistent in HbSS phenotype. Conversely, some studies found no significant association between proteinuria and sickle cell disease [24]. However the inconsistencies in reported findings with proteinuria and SCD, significant proteinuria (which is also the most common presentation in childhood) is a key factor for CKD progression [32].

The principal factors which influence serum creatinine levels are muscle mass and GFR. Whereas children with HbSS had the highest prevalence of CKD, the serum creatinine levels were lower than expected compared to HbAS and HbSC phenotypes. These findings are consistent with previous findings [33–35]. These findings could be attributed to the high prevalence of underweight among the study children especially HbSS. Malnutrition is associated with lower creatinine levels and also a risk factor for CKD which could explain the findings of this study. High urea level designates renal dysfunction [36]. However, the levels of urea observed in this study were lower in HbSS compared with HbAS. Effective dosage of hydroxyurea is known to induce nephro-protective effect for SCD; and this is a probable explanation to the reduced levels of creatinine and urea among HbSS patients compared to Hb AS patients [37].

Various studies have showed that calculation of eGFR using serum creatinine is one of the best markers for predicting kidney disease progression [13, 38, 39]. Alvarez et al., [37] indicated that eGFR for defining CKD in Children (CKD) with Schwartz formula is the best non-invasive method. Also, other studies combine the use of eGFR and albuminuria as the best diagnostic tool for evaluating the progression of renal disease [36, 40]. In this study, the use of KDIGO criteria for CKD classification detected 39.6% of the children having reduced GFR. This results is similar to a cross-sectional study in Ghana that observed a CKD prevalence of 39.2% among SCD children [28]. Aside the findings of the study, the study has some limitations. The prevalence of malnutrition among the children could underestimated the eGFR calculation, hence CKD determination. Also, dipstick method was used for proteinuria evaluation without a second confirmation by spot test or albumin quantitation. Thus, the possibilities of false positive results. However, evaluation of urinary findings was interpreted with caution and expertise knowing that the dipstick method was the most available routinely used method for proteinuria estimation in Ghana. Also, malnutrition is a common feature among SCD children in Ghana as indicated earlier. Generally, consistent with literature, this study has shown that renal abnormalities is associated with children with sickle cell disease.

### Conclusion

This study observed proteinuria, granular cast and chronic kidney disease to be prevalent in the proportions of 26.4%, 5.6% and 39.6%, respectively among sickle cell positive children. The indices of renal abnormalities were most prevalent among HbSS phenotype, then HbSC phenotype. Considering highly resource limited settings, the study findings informs possible renal abnormalities among children with SCD. Hence, renal assessment using routinely available dipstick and urine concentration findings should be prioritize by pediatricians for early detection and evaluation.

## Supporting information

S1 Table Urine dipstick and Microscopic findings. (DOCX)
