# Supplementary material for: Renal abnormalities among children with sickle cell conditions in highly resource-limited setting in Ghana
Source: PLoS One. 2019 Nov 19;14(11):e0225310. doi: 10.1371/journal.pone.0225310 (PMC6863548; doi:10.1371/journal.pone.0225310)
Supplement: S1 Table — (DOCX) [file pone.0225310.s001.docx]

**S1 Table. Urine dipstick and Microscopic findings**

| **Urine Characteristics** | **Total**  **(N=212)** | **Hb variant** | | |  |
| --- | --- | --- | --- | --- | --- |
|  |  | **AS (N=96)** | **SC (N=48)** | **SS (N=68)** | **p-value** |
| **pH** | 5.91±0.45 | 5.95±0.40 | 5.90±0.36 | 5.85±0.55 | 0.403 |
| **Specific gravity** | 1.018±0.007 | 1.017±0.007 | 1.019±0.007 | 1.017±0.007 | 0.233 |
| **Blood** |  |  |  |  | 0.447 |
| Negative | 204 (96.2) | 94 (97.9) | 46 (95.8) | 64 (94.1) |  |
| Positive | 8 (3.8) | 2 (2.1) | 2 (4.2) | 4 (5.9) |  |
| **Nitrite** |  |  |  |  | NA |
| Negative | 212 (100.0) | 96 (100.0) | 48 (100.0) | 68 (100.0) |  |
| Positive | 0 | 0 | 0 | 0 |  |
| **Leukocyte** |  |  |  |  | 0.746 |
| Negative | 185 (87.3) | 82 (85.4) | 43 (89.6) | 60 (88.2) |  |
| Positive | 27 (12.7) | 14 (14.6) | 5 (10.4) | 8 (11.8) |  |
| **Ketones** |  |  |  |  | 0.874 |
| Negative | 200 (94.3) | 90 (93.8) | 46 (95.8) | 64 (94.1) |  |
| Positive | 12 (5.7) | 6 (6.3) | 2 (4.2) | 4 (5.9) |  |
| **Semi-quantitative Findings** |  |  |  |  | *<0.0001* |
| Albuminuria <0.3g/L | 156 (73.6) | 92 (95.8) | 28 (58.3) | 36 (52.9) |  |
| Albuminuria ≥0.3g/L | 56 (26.4) | 4 (4.2)^a^ | 20 (41.7)^b^ | 32 (47.1)^b^ |  |
| **Pus cell per HPF** |  |  |  |  | 0.053 |
| 1-7 (normal) | 152 (71.1) | 76 (79.2) | 29 (60.4) | 47 (69.1) |  |
| >7 (increased) | 60 (28.3) | 20 (20.8) | 19 (39.6) | 21 (30.9) |  |
| **Epithelial cell per HPF** |  |  |  |  | *0.002* |
| **<10** | 202 (95.3) | 96 (100.0) | 46 (95.8) | 60 (95.3) |  |
| 10-15 | 10 (4.7) | 0^a^ | 2 (4.2)^a,b^ | 8 (11.8)^b^ |  |
| **Red blood cell per HPF** |  |  |  |  | 0.253 |
| **0-2** | 196 (92.5) | 90 (93.8) | 46 (95.8) | 60 (88.2) |  |
| **>2** | 16 (7.5) | 6 (6.3) | 2 (4.2) | 8 (11.8) |  |
| **Granular Cast per** |  |  |  |  | *0.002* |
| Not seen | 200 (94.3) | 96 (100.0) | 44 (91.7) | 60 (88.2) |  |
| Seen | 12 (5.7) | 0^a^ | 4 (8.3)^b^ | 8 (12.8)^b^ |  |
| **Crystals** |  |  |  |  | NA |
| Not seen | 212 (100.0) | 96 (100.0) | 48 (100.0) | 68 (100.0) |  |
| Seen | 0 | 0 | 0 | 0 |  |

***HPF- high power field; NA- not available. Italised values represents statistically significant values. “b” is significantly greater than “a”.***
